# Remote sensing-based evapotranspiration and soil water balance estimation for a tropical pasture in Brazil using the SETMI model

**DOI:** 10.1007/s00484-026-03255-9

**Published:** 2026-06-30

**Authors:** Vitor de J. M. Bianchini, Ivo Z. Gonçalves, Christopher M. U. Neale, Alex da S. Sechi, Thieres G. F. da Silva, Fábio R. Marin

**Affiliations:** 1 Department of Biosystems Engineering, Luiz de Queiroz College of Agriculture, University of São Paulo, Piracicaba, SP Brazil; 2 Daugherty Water for Food Global Institute, University of Nebraska-Lincoln, Lincoln, NE USA; 3 Department of Agricultural Engineering, Federal Rural University of Pernambuco, Recife, PE Brazil

**Keywords:** Eddy covariance, Satellite imagery, Vegetation index, Basal crop coefficient, Water productivity, Elephant grass

## Abstract

Developing strategies to improve pasture yield and mitigate environmental impacts is crucial in Southeastern Brazil, where livestock farming faces competition for areas with profitable crops. Accurate evapotranspiration (ET) estimation is an essential component of managing soil water balance and evaluating pasture response to drought and water productivity. The objectives of this study were to: (1) develop a relationship between the basal crop coefficient (K_cb_) derived from observed ET, and the soil-adjusted vegetation index (SAVI) from PlanetScope images; (2) integrate the K_cb_-SAVI relationship into remote sensing-based soil water balance (RSWB) to generate daily K_cb_ and then simulate the actual evapotranspiration (ET_a_) of an intensively grazed tropical pasture in the state of São Paulo, Brazil; and (3) use the spatialized estimate of ET_a_ to assess the crop water productivity (${\mathrm{W}\mathrm{P}}_{{\mathrm{E}\mathrm{T}}_{\mathrm{a} \mathrm{m}\mathrm{o}\mathrm{d}}}$). The K_cb_-SAVI relationship developed in this study showed a strong positive correlation between SAVI and K_cb_, with Pearson correlation coefficient ($\rho$) and ${\mathrm{R}}^{2}$ values of 0.89 and 0.79, respectively. Comparisons between observed and simulated ET_a_ indicated good agreement for daily values ($\mathrm{R}\mathrm{M}\mathrm{S}\mathrm{E}$ of 0.59 mm d^−1^, Willmott index of agreement ($\mathrm{D}$) of 0.86) and for average weekly values ($\mathrm{R}\mathrm{M}\mathrm{S}\mathrm{E}$ of 0.38 mm d^−1^, $\mathrm{D}$ of 0.93). Biases of -5% and 3% were obtained for modelled cumulative ET_a_ in the years 2021–2022 and 2022–2023, respectively. The average ${\mathrm{W}\mathrm{P}}_{{\mathrm{E}\mathrm{T}}_{\mathrm{a} \mathrm{m}\mathrm{o}\mathrm{d}}}$ values were similar for both years, 2.2 kg m^−3^ in 2021–2022 and 1.8 kg m^−3^ in 2022–2023. These findings demonstrate the potential of RSWB, ET_a_, and WP assessments to enhance decision-making, monitoring, and management of water resources in pasture-based livestock farming.

**Supplementary Information:**

The online version contains supplementary material available at 10.1007/s00484-026-03255-9.

## Introduction

The Brazilian livestock sector significantly contributes to global animal-based food production, with a cattle herd of approximately 238.6 million head (IBGE 2023), primarily fed by an estimated 164.3 million hectares of pastures, which represent the predominant land use in Brazil, covering 19.3% of the national territory (MapBiomas 2023a). Brazil is the world’s second-largest beef producer, behind the United States, and the leading exporter, contributing 25% of all global beef exports (USDA-FAS 2025). Additionally, Brazil holds the position of the fifth largest milk producer worldwide (USDA-FAS 2024). The Southeastern Region of Brazil, where our study site was situated, comprises 17% of the national pasturelands (MapBiomas 2023b) and contributes significantly to 33.6% of milk production (Hott et al. 2024) and 22.5% of cattle slaughter (Malafaia and Biscola 2025).

Advancements in pasture-based systems that enhance milk and beef production have been crucial in meeting the increasing demand for animal-based proteins (van Dijk et al. 2021) and mitigating rising production costs following the COVID-19 pandemic. Despite this progress, most pasture-based livestock systems remain inefficient, producing only 20 to 30% of their potential (Marin et al. 2022; dos Santos et al. 2024). The need to halt the expansion of the Brazilian agricultural frontiers (Strassburg et al. 2014; Marin et al. 2022), combined with the pressure caused by the growth of cropland areas at the expense of low-productive pastures (Dias et al. 2016; Parente et al. 2017, 2019; MapBiomas 2023a), along with the high level of pasture degradation, low yields of forage dry matter (de Oliveira Silva et al. 2018), and the greater susceptibility of pastures to climate change (da Silva et al. 2017), highlights the urgency of developing sustainable management practices that minimize the negative environmental impacts associated with Brazilian livestock. To achieve this goal, modelling crop development, soil water balance, and evapotranspiration (ET) estimates are crucial for effectively assessing drought conditions and supporting decision-making to improve forage production management (Andrade et al. 2016).

The ET connects the ecosystem’s water, energy, and carbon cycles (Fisher et al. 2017; da Rocha et al. 2022), and is a key element of the overall water balance (da Silva et al. 2017; Bhatti et al. 2020). For pastures, ET serves as an indicator of how ecosystems respond to rainfall variability and drought events (Alves et al. 2021, 2022; Bezerra et al. 2022; da Silva et al. 2024), and is crucial for evaluating crop water productivity. Furthermore, it plays a key role in monitoring and promoting more efficient management of water resources amid global climate change (Khorchani et al. 2024; D’Acunha et al. 2024). In recent decades, numerous methods have been developed and used to measure ET, including lysimeters, eddy covariance (EC), Bowen ratio, sap flow, and scintillometry. These methods, however, require rigorous experimental care and offer limited spatial coverage (Allen et al. 2011). To overcome these limitations, satellite remote sensing methods (RS) for estimating ET have been widely adopted as a cost-effective solution, enhancing the limited coverage of the Earth’s surface provided by the traditional ground-based observation methods (Wagle et al. 2017).

The RS methods commonly used to estimate ET can be categorized into two main classes. The first class includes models based on surface energy-balance (SEB) approaches, using shortwave, near-infrared and thermal band inputs to calculate net radiation, soil heat flux, and sensible heat flux. Latent heat flux and ET are then calculated as an energy balance equation residual (Neale et al. 2012). The second class includes models that estimate ET from reflectance-based basal crop coefficients (K_cbrf_), which do not use thermal band inputs and are designed from a simple linear relationship between basal crop coefficients (K_cb_) obtained from field measurements and spectral vegetation indices (VI) (Neale et al. 1989) derived from remotely sensed canopy reflectance data. This study adopted the K_cbrf_-based method implemented in the SETMI model (Spatial Evapotranspiration Modeling Interface) (Neale et al. 2012). This method monitors crop growth using a VI from a time series of satellite images. The VI is applied for K_cbrf_ estimation at the satellite overpass and adjusts the daily K_cb_ curve over time to reflect growing conditions. To obtain the actual evapotranspiration (ET_a_), the K_cbrf_ values are integrated into a RSWB model, following the approach described by Allen et al. (1998) (Bispo et al. 2022).

Reflecting the importance of livestock in Brazil and the role of pasture intensification for sustainable food production, this study aimed to estimate the ET and soil water balance using the SETMI model in an area of intensive production to support forage management decision-making in pasture-based livestock systems. SETMI requires a specific K_cb_-VI relationship for vegetation cover under the climatic condition of the target environment to accurately estimate ET and soil water balance. Since a K_cb_-VI relationship for intensively managed tropical pastures has not been developed so far, this study set out to create it using EC data and high spatial and temporal resolution images. Upon creating the K_cb_-VI relationship and parameterizing SETMI, we used the model to assess the daily K_cb_ and ET_a_ and simulate the RSWB for two consecutive years in Southeastern Brazil, a key region for tropical livestock intensification.

## Material and methods

### Study site

**Fig. 1:**
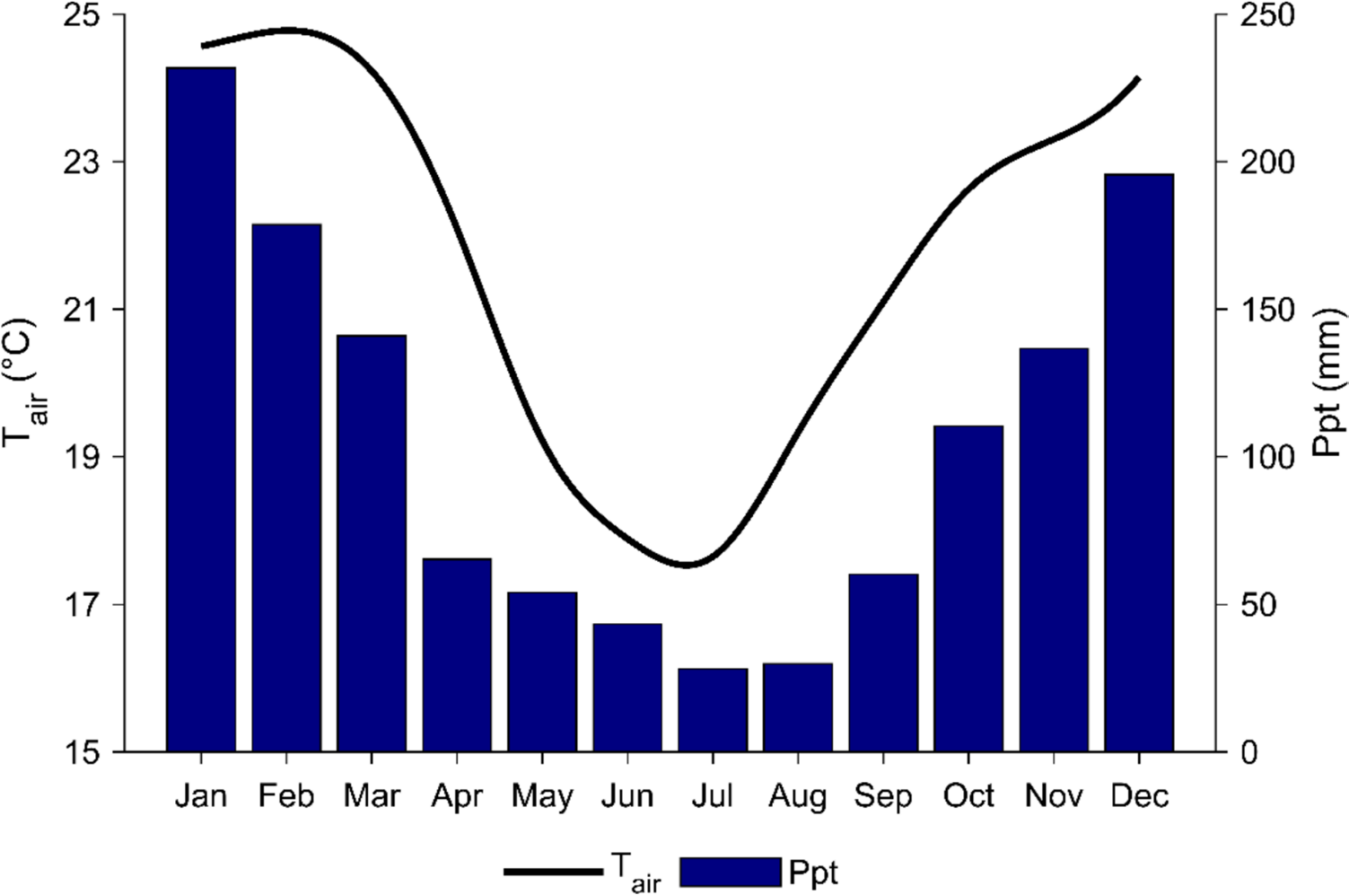
Monthly average precipitation (Ppt) and air temperature (T_air_) based on the climatological normal for the period 1917–2024 in the study region

The study took place at the Luiz de Queiroz College of Agriculture (Esalq) of the University of São Paulo (USP) in the city of Piracicaba, State of São Paulo, Brazil (22°42′S, 47°38′W and 546 a.s.l). The climate of Piracicaba is tropical with a dry winter (Aw), based on the Köppen climate classification (Peel et al. 2007; Alvares et al. 2022) and is marked by two well-defined seasons, a hot and wet summer, and a mild and dry winter. The highest volumes of rainfall are usually recorded between December and February, while the driest period extends from April to September. Fall and spring are considered transitional seasons. The average annual temperature and precipitation are recorded at 21.7 ºC and 1275 mm. Figure 1 shows the monthly average precipitation (Ppt) and air temperature (T_air_) based on the climatological normal for the period 1917–2024 in the study region (http://www.leb.esalq.usp.br/leb/postoaut.html).

The experimental site cover consisted of rainfed elephant grass (*Pennisetum purpureum* Schum. cv. Cameroon) pasture, established on Eutroferric Red Nitosol (Rhodic Kandiudalf) (Santos et al. 2018) in 1972 and has been grazed intermittently by dairy cows (Batalha et al. 2022). Elephant grass is a C4 tropical perennial grass known for its tussock-forming growth habit, high CO_2_ assimilation efficiency, and substantial herbage accumulation under optimal growth conditions (Pereira et al. 2013; Botero-Londoño et al. 2021; de Oliveira Gonçalves et al. 2022).

### Pasture management

The pasture was divided by electric fences into paddocks of approximately 0.20 hectares and subjected to strategies of intermittent stocking management (i.e., rotational grazing management). The sward surface height was the parameter used to monitor the sward structure. Grazing management followed a pre-grazing sward surface height target of 1.00 m, in which the canopy of the elephant grass sward intercepts 95% of the incident radiation (Voltolini et al. 2010; Congio et al. 2018). Each paddock’s pre- and post-grazing sward surface heights were measured from the soil surface to the sward top using a centimeter-graduated stick (Voltolini et al. 2010; Pereira et al. 2014a; Congio et al. 2018). Grazing was performed by lots of Holstein dairy cows of 20 to 25 animals, maintaining a stocking rate of approximately eight cows ha^−1^ during the wet season. During the dry season, when the herd’s nutritional demand exceeded the herbage accumulation, they were kept in a free-stall barn, substantially reducing the grazing frequency.

### Evapotranspiration measurements

Measurements of turbulent fluxes were performed using the EC method from June 20, 2021 to June 02, 2023. The EC system consisted of an IRGASON (Campbell Scientific Inc., Logan, UT, USA), an integrated open-path CO_2_/H_2_O gas analyzer, and a 3D sonic anemometer. Data on H_2_O concentration, u_x_, u_y_, u_z_, and sonic temperature were stored at a 20 Hz sampling rate on a data logger (CR1000, Campbell Scientific Inc., Logan, UT, USA). The EC system was mounted on a mast 2.50 m above ground, high enough for the sensors to remain more than 1.00 m above the sward surface during its maximum development.

Meteorological variables were measured to support the analysis of turbulent fluxes. Net radiation was measured using a net radiometer (CNR4, Kipp & Zonen, Delft, the Netherlands), relative humidity and air temperature with a Vaisala probe (HMP155, Vaisala Corporation, Helsinki, Finland), precipitation with a tipping bucket rain gauge (TB4MM, Campbell Scientific Inc., Logan, UT, USA), and soil heat flux with heat flux plates (HFP01SC-L, Hukseflux Thermal Sensors BV, Delft, Netherlands). We installed the CNR4 and HMP155 sensors 2 m above the ground, the TB4MM sensor 1.5 m above the ground, and the heat flux plates 0.03 m below the soil surface.

Turbulent energy fluxes were calculated for each 30-min interval using EddyPro 7.0.9 software (LI-COR Biosciences, Lincoln, NE, USA). Flux data were filtered using the quality flags (qc) based on two tests known as the steady state test and the developed turbulent conditions test. These qc values ranged from 0 (best quality) to 2 (poor), and qc values equal to 2 were discarded (Foken et al. 2004). A footprint analysis was conducted (Kljun et al. 2015) (Supplementary Fig. S1), and any data periods where less than 80% of the footprint fell within the field of interest were excluded. Calculated fluxes also were excluded during rainfall events and half an hour afterwards due to malfunction of the EC system. The remaining inconsistent values were detected and eliminated by comparing the half-hour flux with the 200-point moving mean and standard deviation (Béziat et al. 2009). Data gaps were filled in using the REddyProc online tool (Max Planck Institute for Biogeochemistry, Germany) (Wutzler et al. 2018).

We evaluated the energy balance closure as a proxy of the EC flux measurements according to Majozi et al. (2017) and observed consistency with results from other EC sites globally. Using the ordinary least squares method, the linear regression between turbulent fluxes (H + LE) and available energy (Rn-G), we obtained a slope of 0.72, an intercept of 13.25, and a coefficient of determination of 0.93 for half-hourly periods; a slope of 0.77, an intercept of 7.52, and a coefficient of determination of 0.94 were obtained for daily periods. The ratio of the turbulent flux term to the available energy term was about 0.84 and 0.83 for half-hourly and daily periods, respectively. Additionally, following Consoli et al. (2014) and Jin et al. (2017), we did not use any technique to force the closure of the energy balance. Finally, the actual evapotranspiration (ET_a_) (mm d^−1^) was calculated as described by Ding et al. (2010).

### Spatial evapotranspiration modeling interface

We created a K_cb_-VI relationship for cultivated elephant grass under intensive grazing in tropical climate. For this purpose, the SAVI (Huete et al. 1985; Huete 1988) was estimated using red (650—680 nm) and near-infrared (845—885 nm) multispectral bands from PlanetScope images with high spatial and temporal resolution over two years (2021–2022 and 2022–2023). SAVI was calculated pixel-by-pixel and averaged at the paddock scale, ensuring that border pixels were excluded. One hundred twenty-five cloud-free surface reflectance images were downloaded from the PlanetScope platform (https://developers.planet.com) for the evaluated period, of which 78 were used in the K_cb_-VI relationship.

K_cb_ was estimated using ET_a_ measured by the EC system (ET_a meas_) and reference evapotranspiration (ET_0_) estimated through the FAO-Penman–Monteith method (Allen et al. 1998) with data from the Esalq/USP weather station (http://www.leb.esalq.usp.br/leb/postoaut.html), located about 200 m from the study site. The dates selected to create the K_cb_-VI relationship were set at least four days after precipitation events, thus ensuring that the soil surface was dry, and the water supply did not restrict transpiration (Bispo et al. 2022). Under these conditions, the value of K_c_ is equivalent to that of K_cb_.

SETMI uses the regression method proposed by Campos et al. (2017) and implemented by Barker et al. (2018) and Bispo et al. (2022) to generate daily SAVI values based on accumulated growing degree-day (base temperature equal to 10 ºC (Singh et al. 2013)). After estimating the daily SAVI and applying the K_cb_-SAVI relationship, the daily K_cb_ can be estimated.

The RSWB model implemented in SETMI, described in detail by Neale et al. (2012), Campos et al. (2017), and Barker et al. (2018), generally follows the soil water balance proposed in the FAO-56 manual (Allen et al. 1998) with enhancements to simulate evaporation from the topsoil layer and account for the temporal evolution of K_cbrf_ (Campos et al. 2017). We used a maximum root zone depth of 0.60 m (Huot et al. 2020; Gonçalves et al. 2024). The depletion factor used was 0.60 according to Allen et al. (1998). We also assumed an evaporative layer depth of 0.05 m (Barker et al. 2018). The daily water uptake from the soil layers was estimated from pasture modeled ET (ET_a mod_) (Neale et al. 2012), which was calculated using ET_0_ and a dual crop coefficient (Allen et al. 1998). The values of ET_a mod_ directly depend on the accuracy of the RSWB model in predicting the soil water content deficit coefficient (K_s_) and soil surface evaporation coefficient (K_e_) (Campos et al. 2017).

**Fig. 2:**
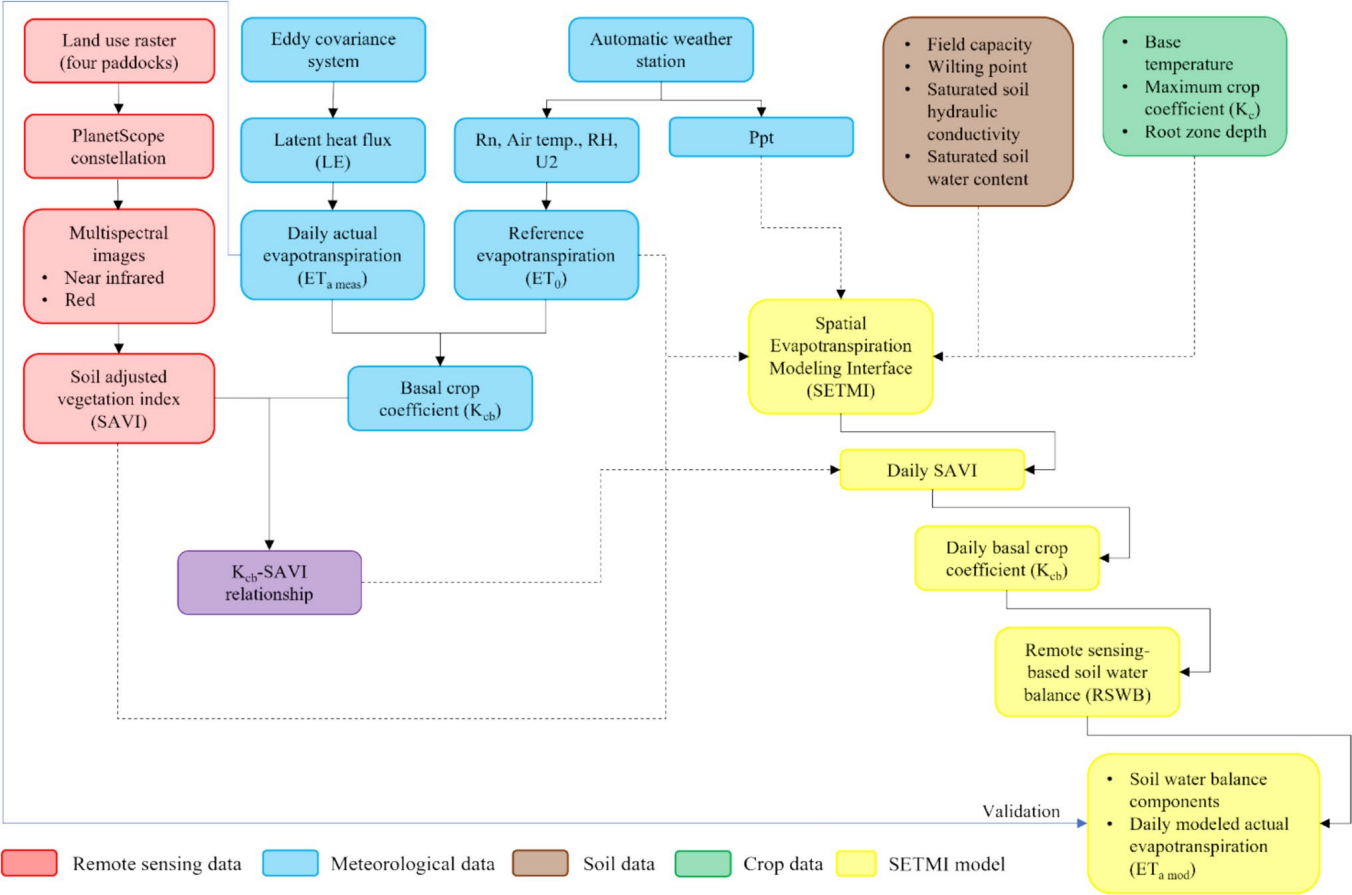
Flowchart demonstrating how evapotranspiration was assessed using the K_cbrf_-based method implemented in the SETMI model

SETMI incorporates the physical properties of three distinct soil layers along the profile (Table 1) to enhance result accuracy (Barker 2017). The saturated soil hydraulic conductivity (469 mm day^−1^) and saturated soil water content (0.46 cm^3^ cm^−3^) were determined by da Silva et al. (2007). The effective precipitation available in SETMI was estimated using the SCS runoff equation formulated by the U.S. Soil Conservation Service (USDA-NRCS 2004; Barker 2017). Figure 2 presents a flowchart detailing the step-by-step process for estimating daily ET_a mod_ and validating the model.

**Table 1:** Physical properties of the soil by depth layer in the study site (Gonçalves et al., (2024)

	Soil layer		
	0—0.2 m	0.2—0.4 m	0.4—0.6 m
Sand (%)	35.7	29.3	25.6
Silt (%)	19.2	18.7	13.4
Clay (%)	45.1	52.0	61.0
Ө_FC_ (cm^3^ cm^−3^)	0.39	0.38	0.42
Ө_WP_ (cm^3^ cm^−3^)	0.29	0.29	0.34

*Ө*_*FC*_ volumetric moisture at field capacity; *Ө*_*WP*_ volumetric moisture at the permanent wilting point

### Crop measurements

The crop measurements were conducted in four representative paddocks (Supplementary Fig. S1) immediately before and after each grazing event, following the rotation of the herd among the paddocks. Non-destructive measurements were performed with the LAI 2200 (LI-COR Inc., Lincoln, NE, USA) to determine the leaf area index (LAI). Pre- and post-grazing herbage mass was measured in each grazing cycle using five randomly collected square samples (4 m^2^ each) from each paddock (Congio et al. 2018). The herbage was cut at ground level, weighed when fresh, and a sub-sample was taken to the laboratory. The sub-sample was manually separated into plant-part components: leaf (leaf blades), stem (stems and leaf sheaths), and dead material, and then dried in a forced-air dryer at 65 °C to a constant weight (Congio 2018).

### Crop water productivity

Crop water productivity based on the ${\mathrm{E}\mathrm{T}}_{\mathrm{a} \mathrm{m}\mathrm{o}\mathrm{d}}$ (${\mathrm{W}\mathrm{P}}_{{\mathrm{E}\mathrm{T}}_{\mathrm{a} \mathrm{m}\mathrm{o}\mathrm{d}}}$) (kg m^−3^) was calculated for the growing seasons of 2021–2022 and 2022–2023 following Eq. 1:

1 $${\mathrm{W}\mathrm{P}}_{{\mathrm{E}\mathrm{T}}_{\mathrm{a} \mathrm{m}\mathrm{o}\mathrm{d}}}= \frac{\mathrm{D}\mathrm{M}\mathrm{F}}{10\times {\mathrm{E}\mathrm{T}}_{\mathrm{a} \mathrm{m}\mathrm{o}\mathrm{d}}}$$

where $\mathrm{D}\mathrm{M}\mathrm{F}$ is the dry matter of leaves accumulated between grazing cycles (kg ha^−1^), and ${\mathrm{E}\mathrm{T}}_{\mathrm{a} \mathrm{m}\mathrm{o}\mathrm{d}}$ is the modeled actual evapotranspiration (mm).

### Statistical analysis

The K_cb_-VI linear relationship was evaluated using the Pearson correlation coefficient ($\rho$) (Pearson 1896) and the coefficient of determination (${\mathrm{R}}^{2}$) (Zou et al. 2003). To verify the predictive accuracy of the SETMI model for estimating evapotranspiration, daily values, and weekly averages, we computed the ${\mathrm{R}}^{2}$, the root mean squared error ($\mathrm{R}\mathrm{M}\mathrm{S}\mathrm{E}$), the index of agreement ($\mathrm{D}$) (Willmott and Wicks 1980; Willmott 1981), and the bias variation ($\mathrm{B}$). We also used the $\mathrm{B}$ to compare the annual cumulative values of ET_a mod_ and ET_a meas._

## Results

### Meteorological conditions

**Fig. 3:**
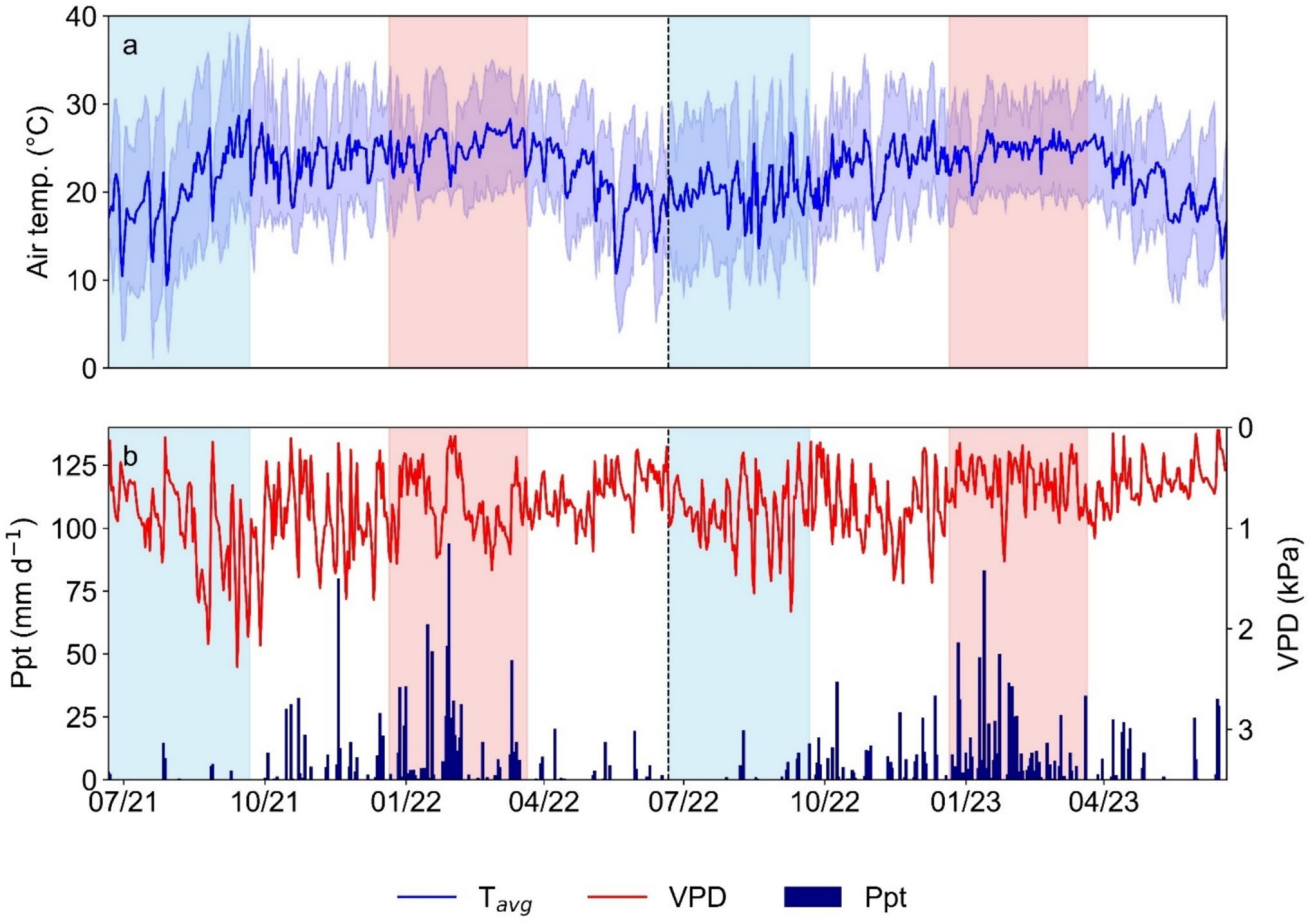
**a** Daily average air temperature (T_avg_) and **b** daily gross precipitation (Ppt) and vapor pressure deficit (VPD). The blue-shaded regions represent the winters, and the red-shaded regions the summers. The unshaded (white) regions correspond to the transitional seasons: spring and fall. The vertical dashed line separates the 2021–2022 (left) and 2022–2023 (right) periods

The average daily temperature (T_avg_), daily gross precipitation (Ppt), and the average daily vapor pressure deficit (VPD) for the years 2021–2022 and 2022–2023 are shown in Fig. 3. In the year 2021–2022, the T_avg_ was 22.5 °C and in the year 2022–2023 it was 22.1 °C, values higher than the historical average (21.7 °C). During the experiment, the T_avg_ was higher in all seasons of 2021–2022 (Supplementary Table S1), especially in spring and summer, when the T_avg_ was 23.7 and 25.2 °C (Supplementary Table S1), exceeding the average values for the same periods in the following year by 0.7 and 0.5 °C. Precipitation was more abundant in 2022–2023 when the total accumulated volume was 1403 mm (Supplementary Table S1), which was 16% higher than the previous year and 10% above the historical average (1275 mm). In 2021–2022, approximately 88% of the rainfall occurred in summer and spring, while in the following year, around 79% of the total rainfall volume was concentrated in these seasons. On average, VPD values were 17% lower in 2022–2023, with a notable decrease during the summer, when the atmospheric evaporative demand was approximately 29% lower than in the summer of 2021–2022 (Supplementary Table S1).

### Relationship between SAVI, K_cb_ and LAI 

**Fig. 4:**
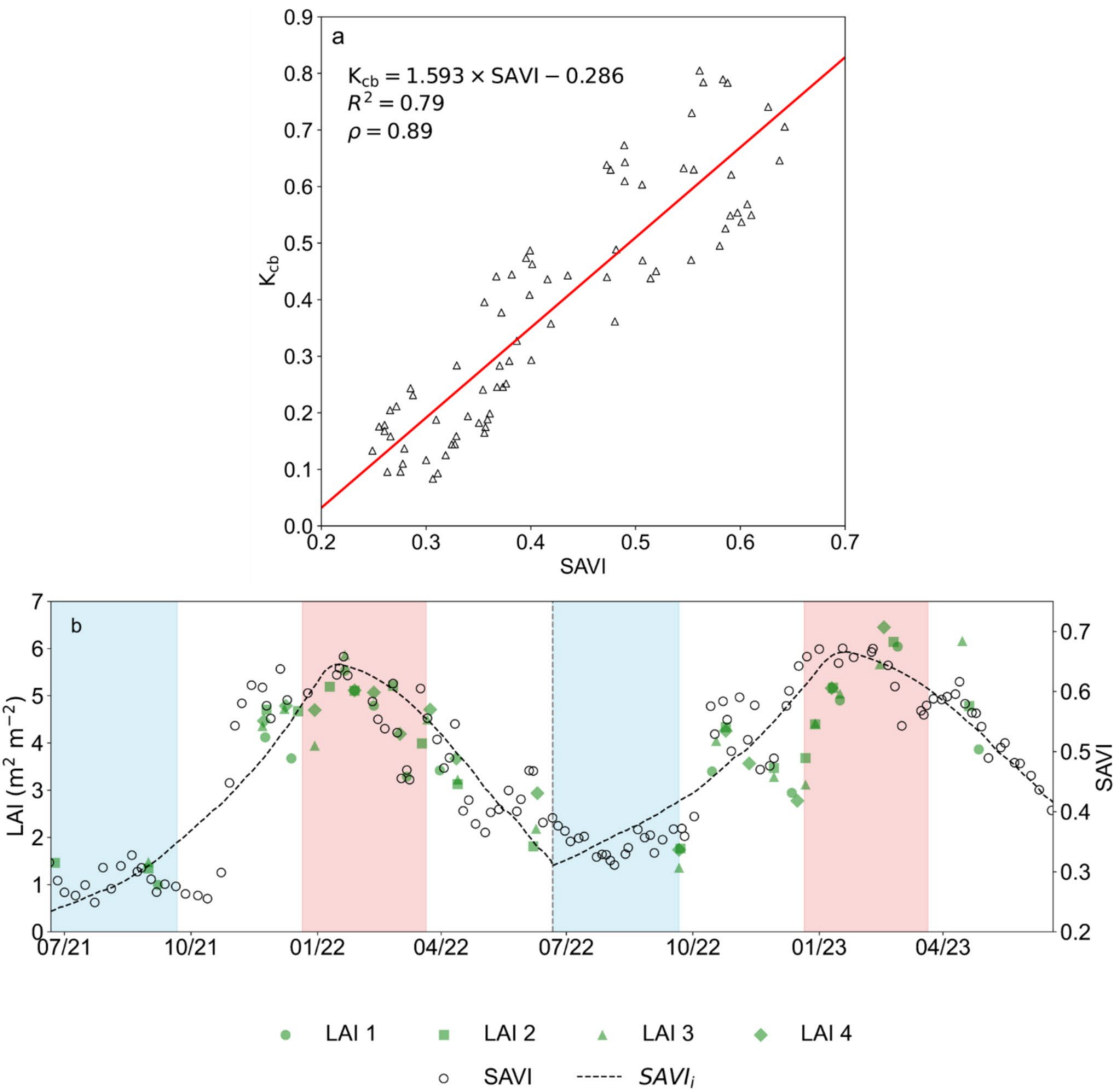
**a** Correlation between the basal crop coefficient (K_cb_) and soil-adjusted vegetation index (SAVI) for a cultivated and intensively grazed tropical pasture of elephant grass. **b** Progression of the leaf area index (LAI) and the soil-adjusted vegetation index (SAVI). LAI 1 to LAI 4 represent the LAI measured in each of the four evaluated paddocks. *SAVI*_*i*_ corresponds to the interpolated values of SAVI. The blue-shaded regions represent the winters, and the red-shaded regions the summers. The unshaded (white) regions correspond to the transitional seasons: spring and fall. The vertical dashed line separates the 2021–2022 (left) and 2022–2023 (right) periods

SETMI uses a specific K_cbrf_ derived from a linear relationship that incorporates K_cb_ values from observed ET data and SAVI values obtained through remote sensing. Figure 4a shows the K_cb_-SAVI relationship developed for a cultivated and intensively grazed tropical pasture in Brazil, demonstrating a strong positive correlation between SAVI and K_cb_, with $\rho$ and ${\mathrm{R}}^{2}$ values of 0.89 and 0.79, respectively. The K_cb_ values ranged from 0.08 to 0.80, while the SAVI values varied between 0.25 and 0.64.

The SAVI values obtained through remote sensing accurately reflected the pasture’s growth trend (Fig. 4b). The maximum and minimum values of SAVI observed in the year 2021–2022 were 0.69 and 0.24, while in 2022–2023, they were 0.72 and 0.29. In both years, SAVI and LAI reached their maximum and minimum values during the same periods. SAVI values begin to increase during the spring with the onset of the rainy season, reaching their peak in the summer, when forage accumulation intensifies. They then decline during the fall as water availability and solar radiation decrease, eventually reaching their lowest levels in winter.

### Remote sensing-based soil water balance

**Fig. 5:**
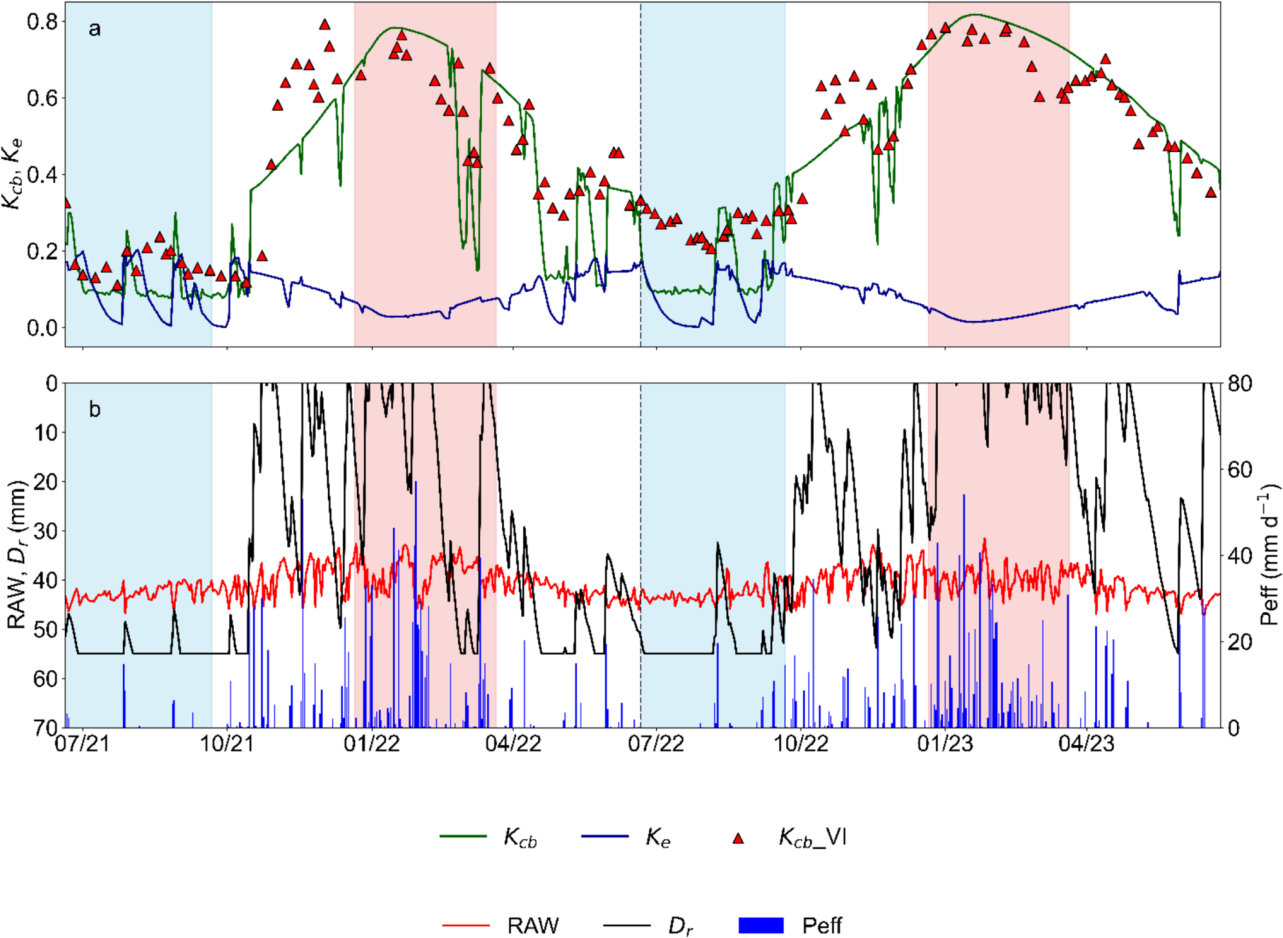
**a** Estimated basal crop coefficient (K_cb_), evaporation coefficient (K_e_), and reflectance basal crop coefficient (K_cb__VI). **b** Readily available water (RAW), water depletion (D_r_), and effective precipitation (Peff). The blue-shaded regions represent the winters, and the red-shaded regions the summers. The unshaded (white) regions correspond to the transitional seasons: spring and fall. The vertical dashed line separates the 2021–2022 (left) and 2022–2023 (right) periods

The SETMI model accurately estimated daily K_cb_ values throughout the two-year period (Fig. 5a) by utilizing interpolated daily SAVI values throughout the year, combined with the K_cb_-SAVI relationship presented in Fig. 4a. The maximum and minimum K_cb_-VI values observed in the year 2021–2022 were 0.75 and 0.10, respectively, while in 2022–2023 they were 0.81 and 0.17. The K_e_ values exhibited an inverse pattern to K_cb_ values, decreasing during wet periods and times of higher LAI and increasing during dry periods and times of lower LAI.

The soil water balance model was applied daily, and the results were validated using ET_a_ measurements obtained by the EC system. Periods of water stress are evident in Fig. 5b where soil water depletion (D_r_) surpasses the readily available water (RAW) for plants. In both years analyzed, the pasture was more affected by water stress during winter and transitional seasons. Additionally, water stress was observed in the late summer of 2021–2022.

### Evapotranspiration assessment and water productivity

The ET_a mod_ values mirrored the trends of ET_0_ values (Supplementary Fig. S2). The cumulative ET_a mod_ was 775 and 821 mm for the first and second years, respectively (Supplementary Table S1). On average, the ET_a mod_ and ET_0_ were 2.1 ± 1.5 and 4.8 ± 2.0 mm d^−1^ in 2021–2022, and 2.3 ± 1.4 and 4.2 ± 1.7 mm d^−1^ in 2022–2023. Larger discrepancies between ET_a mod_ and ET_0_ values were observed during the dry periods, when forage accumulation, SAVI, K_cb_, and LAI values were low, reaching their minimum.

**Fig. 6:**
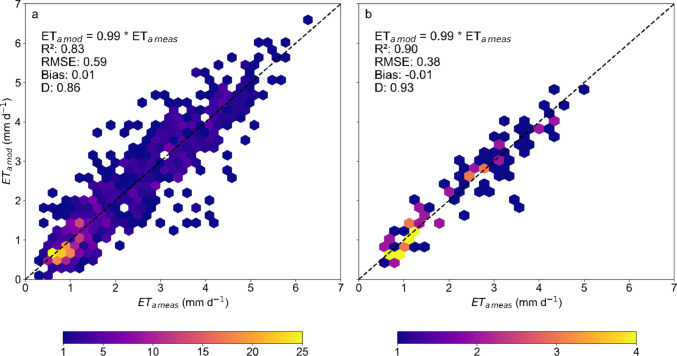
**a** Scatterplots of the daily and **b** weekly averages of measured (ET_a meas_) and modeled (ET_a mod_) actual evapotranspiration for the entire study period. The black dashed lines represent the 1:1 reference line. The bars below the plots represent the data density

Daily and weekly average values of ET_a meas_ and ET_a mod_ were compared using simple linear regression equations (Fig. 6). The values of R^2^, RMSE, B, and D were 0.83, 0.59 mm d^−1^, 0.01, and 0.86 for the daily data comparison and 0.90, 0.38 mm d^−1^, −0.01 and 0.93, respectively, for the weekly average comparison. In both cases, the model showed a B of approximately 1%. Comparing the weekly averages of ET_a_ reduced noise and enhanced the model’s performance. Although some dispersion was observed in the comparison of measured and modeled values, the agreement achieved for the ET_a_ values during the study period supports the use of SETMI model to estimate ET_a_ for managed tropical pastures.

**Fig. 7:**
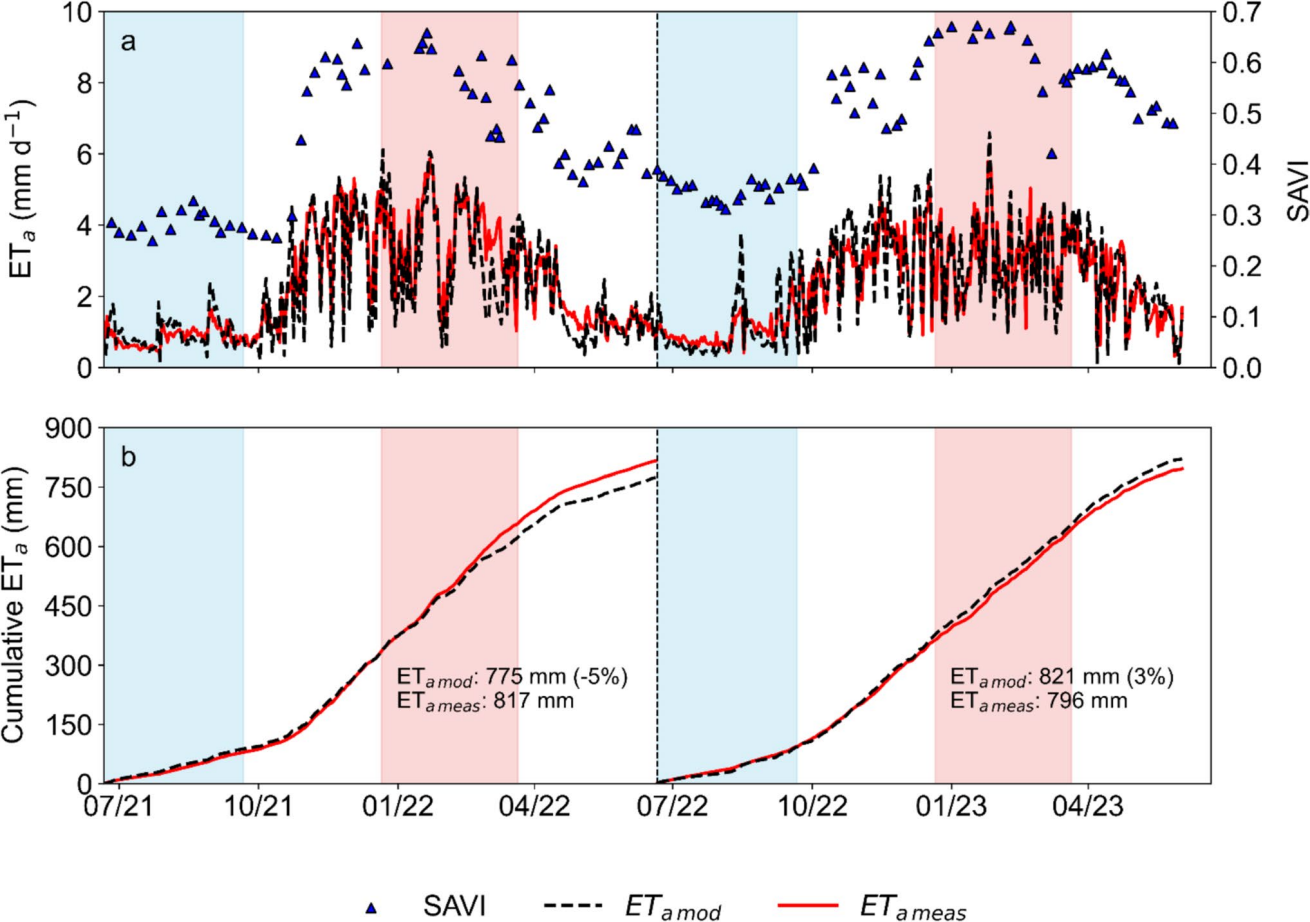
**a** Daily modeled (ET_a mod_) and measured (ET_a meas_) actual evapotranspiration and soil-adjusted vegetation index (SAVI). **b** Cumulative ET_a mod_ and ET_a meas_. The blue-shaded regions represent the winters, and the red-shaded regions the summers. The unshaded (white) regions correspond to the transitional seasons: spring and fall. The vertical dashed line separates the 2021–2022 (left) and 2022–2023 (right) periods

Figure 7 shows the daily variability of ET_a meas_ and ET_a mod_, as well as the cumulative values for both years. The most significant divergence between the measured and simulated values occurred during the summer of 2021–2022, when the cumulative ET_a meas_ exceeded the ET_a mod_ by 11%. This discrepancy also is highlighted in Supplementary Fig. S3, which shows the distribution of modeled and measured ET_a_ values, along with the ET_a_/ET_0_ ratio for all seasons during the two-year period. In the summer of 2021–2022, ET_a mod_ represented an average of 71% of ET_0_, while ET_a meas_ accounted for approximately 84% of ET_0_. During winter, ET_a mod_ and ET_a meas_ generally represented 22% and 24% of ET_0_, respectively.

**Fig. 8:**
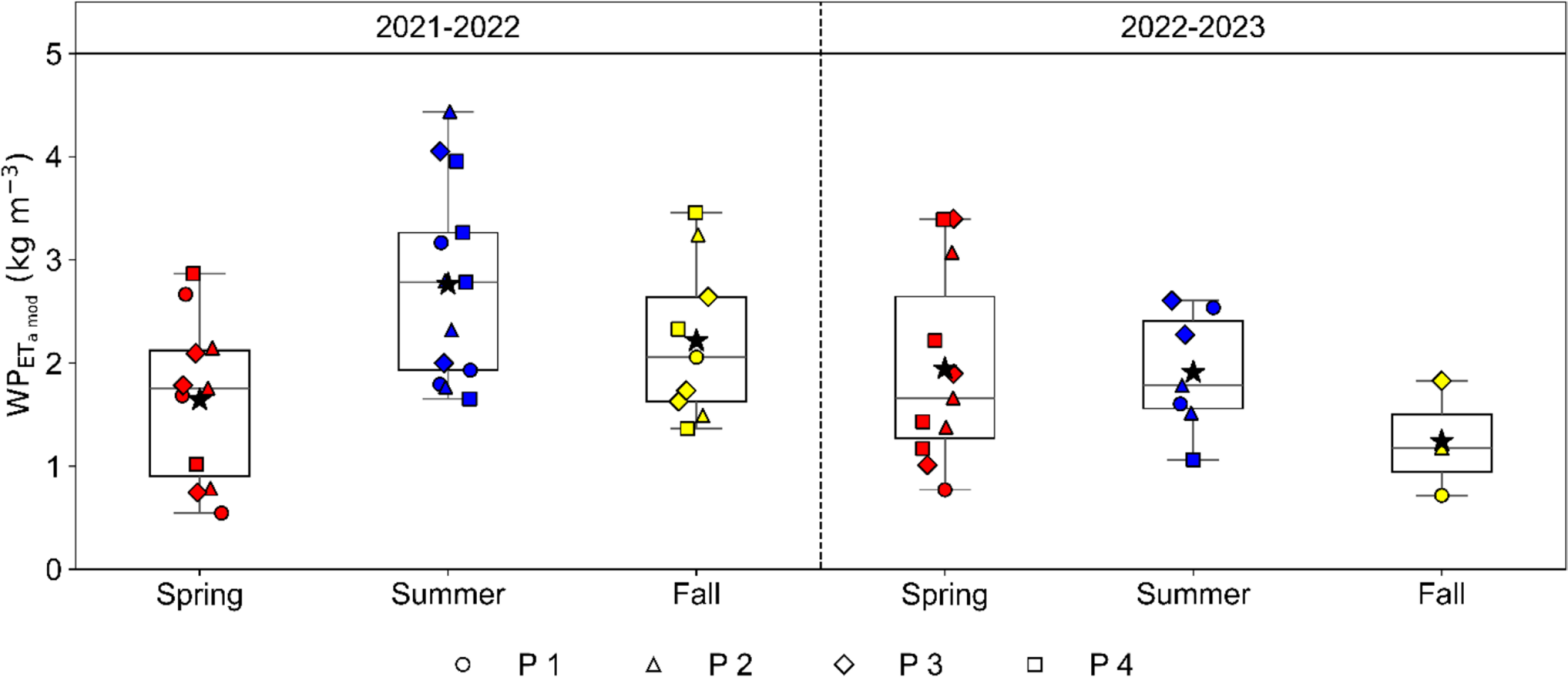
Crop water productivity based on the modeled actual evapotranspiration (${\mathrm{W}\mathrm{P}}_{{\mathrm{E}\mathrm{T}}_{\mathrm{a} \mathrm{m}\mathrm{o}\mathrm{d}}}$) for the growing seasons of 2021–2022 and 2022–2023. P1 to P4 represent the four evaluated paddocks

The distribution of ${\mathrm{W}\mathrm{P}}_{{\mathrm{E}\mathrm{T}}_{\mathrm{a} \mathrm{m}\mathrm{o}\mathrm{d}}}$ values calculated for the years 2021–2022 and 2022–2023 is presented in Fig. 8. Winter was excluded from this analysis due to the low sward growth rates and a reduced number of grazing events. The average ${\mathrm{W}\mathrm{P}}_{{\mathrm{E}\mathrm{T}}_{\mathrm{a} \mathrm{m}\mathrm{o}\mathrm{d}}}$ values were similar for both years, with 2.2 kg m^−3^ in 2021–2022 and 1.8 kg m^−3^ in 2022–2023. In 2021–2022, the highest ${\mathrm{W}\mathrm{P}}_{{\mathrm{E}\mathrm{T}}_{\mathrm{a} \mathrm{m}\mathrm{o}\mathrm{d}}}$ values were obtained in summer, reaching a maximum of 4.4 kg m^−3^. The lowest values for 2021–2022 occurred in spring, with a minimum of 0.5 kg m^−3^. In 2022–2023, the pasture exhibited the same ${\mathrm{W}\mathrm{P}}_{{\mathrm{E}\mathrm{T}}_{\mathrm{a} \mathrm{m}\mathrm{o}\mathrm{d}}}$ during spring and summer (1.9 kg m^−3^). However, unlike the previous year, the maximum and minimum ${\mathrm{W}\mathrm{P}}_{{\mathrm{E}\mathrm{T}}_{\mathrm{a} \mathrm{m}\mathrm{o}\mathrm{d}}}$ values were recorded in spring (3.4 kg m^−3^) and fall (0.7 kg m^−3^), respectively.

## Discussion

K_cb_-VI approaches provide a robust and straightforward method for estimating crop evapotranspiration, offering spatially and temporally distributed information throughout the crop cycle and enabling daily interpolation (Pôças et al. 2020). K_cb_-VI relationships are less sensitive to changes in canopy architecture than K_cb_-LAI relationships (Choudhury et al. 1994), as shown by Campos et al. (2017), who found no significant differences in SAVI and K_cb_ peaks between older and newer maize hybrids but observed substantial differences in LAI values. Using VIs to access K_cb_ is particularly beneficial for grazed tropical pastures. Beyond the variations in sward structure among different grass cultivars, various grazing management strategies can significantly impact the herbage accumulation pattern of the same cultivar (Pereira et al. 2013, 2014b, 2014a; Congio et al. 2018). K_cb_-LAI relationships are more specific and determining LAI is labor-intensive, time-consuming, and requires skilled personnel. In contrast, spectral data in the visible and near-infrared ranges can be readily obtained from various satellite missions. To the best of our knowledge, this is the first study to develop a K_cb_-VI relationship for intensively grazed tropical pastures, showing a strong positive correlation between SAVI and K_cb_.

The highest SAVI values were recorded during spring and summer, coinciding with the highest LAI values observed during the experiment and with the periods of greatest net leaf accumulation (Pereira et al. 2014a) (Fig. 4b). An increased frequency of tussocks with larger perimeter and a reduced frequency of bare ground also contributed to the SAVI peak during the summers of both years analyzed (Congio et al. 2018). The lowest LAI and SAVI values were recorded during the winters of 2021-2022 and 2022-2023, as well as the fall of 2021-2022, reflecting reduced pasture growth rates during the seasons characterized by low precipitation and colder temperatures (Supplementary Table S1). The decrease in SAVI during dry periods also was associated with a higher frequency of bare ground and a lower frequency of tussocks with smaller perimeters compared to wet periods (Congio et al. 2018). Elephant grass, being a tropical perennial tussock-forming grass species (Pereira et al. 2013), maintains at least 30% of the soil surface coverage by tussocks after establishment (Pereira et al. 2014b; Congio et al. 2018). For this reason, the minimum SAVI values in this study are higher than those observed in annual crops by Campos et al. (2017) and Neale et al. (2012), where the field was predominantly bare at the start of the crop cycle.

The ET_a mod_ values mirrored the trends of ET_0_ values during the wet periods. In 2021-2022, the rainy season extended from October 2021 to March 2022, while in 2022-2023, it lasted from September 2022 to April 2023. The highest differences between ET_a mod_ and ET_0_ were observed during the winters of 2021-2022 and 2022-2023, the driest seasons (Supplementary Fig. S2). High K_cb_ and low K_e_ values were noted during rainy periods when sward growth rates and LAI values were elevated; the opposite trend was observed during the dry periods (Fig. 5a). Combining the K_cbrf_ approach with the soil water balance model enabled the identification of water stress periods, where low soil water availability limited herbage accumulation. These periods are evident in Fig. 5b when Dr exceeds RAW values. The cumulative water deficit (ET_a mod_ - ET_0_) was 718 mm for 2021-2022 and 395 mm for 2022-2023.

The values of R^2^ and D obtained for both the daily and weekly comparisons indicate that the model is capable of accurately reproducing ET_a meas_ (Fig. 6). The $\mathrm{R}\mathrm{M}\mathrm{S}\mathrm{E}$ values for ET_a_ on a daily scale found in this study (0.59) is comparable to the values reported by Bispo et al. (2022) (0.88) and Gonçalves et al. (2022) (0.76 and 0.62), using a hybrid approach and geeSEBAL, respectively, for sugarcane in São Paulo, Brazil. We observed an improvement in the model’s performance when comparing weekly ET_a_ values. Using weekly average helps minimize the impact of the soil water evaporation model’s lower accuracy for daily estimates and reduces the influence of short-term weather variations on crop evapotranspiration rates (Torres and Calera 2010; Campos et al. 2017). The season in which there was the most significant discrepancy between measured and simulated ET_a_ values was the summer of 2021–2022 (Fig. 7). The model excessively penalized transpiration rates during first ten days of March 2022, a period marked by increased atmospheric water demand, reduced rainfall (Fig. 3), and decreased SAVI and LAI values, which were approximately 0.45 and 3, respectively (Fig. 4b). The cumulative water deficit during the summer of 2021–2022 was 63 mm (Fig. 5b).

Due to their shallower roots, pastures are particularly vulnerable to climate change (da Rocha et al. 2022; Zheng et al. 2023) and more susceptible to severe droughts than forests (Alves et al. 2022). This shallowness leads to a rapid reduction of the soil water content of the upper soil layers, which influences variations in mass and energy fluxes (Meirelles et al. 2011; da Silva et al. 2017, 2024; Alves et al. 2022). In this context, irrigation emerges as a potential solution to extend the forage production season, boost herbage yield, increase stocking rates, and intensify pasture-based tropical livestock systems, thus addressing current limitations and land use efficiencies (Ribeiro et al. 2008; Gonçalves et al. 2024; dos Santos et al. 2024). The SETMI model, utilizing the K_cb_-VI approach and a soil water balance model, can support decision-making by monitoring ET and soil moisture. It also can contribute to the development of public policies for water resource management in pasture-based livestock systems and enhance irrigation management practices.

SETMI’s capability to spatially simulate ET_a_ was employed to evaluate the ${\mathrm{W}\mathrm{P}}_{{\mathrm{E}\mathrm{T}}_{\mathrm{a} \mathrm{m}\mathrm{o}\mathrm{d}}}$ of the studied elephant grass pasture. Although the average ${\mathrm{W}\mathrm{P}}_{{\mathrm{E}\mathrm{T}}_{\mathrm{a} \mathrm{m}\mathrm{o}\mathrm{d}}}$ values obtained for both years were similar, there was a notable difference between the summer average for 2021–2022 and 2022–2023, which were 2.8 and 1.9 kg m^−3^, respectively (Fig. 8). T_avg_, VPD, ET_0_, and ET_a mod_ were lower in the summer of 2022–2023 than the previous year. In addition to environmental conditions, grazing management may have interfered with this result. In the summer of 2022–2023, the defoliation frequency and grazing intensity in the pdaaddocks evaluated were reduced, leading to the higher LAI values (Fig. 4b). LAI and light interception values above the recommended maximum for tropical grasses are linked to a lower leaf accumulation rate (da Trindade et al. 2007; Pereira et al. 2013, 2014a; da Silva et al. 2015; Congio et al. 2018) and a greater proportion of mature leaves with low photosynthetic efficiency (Wilson and Kennedy 1996).

## Conclusions

- This study was conducted in a key region of livestock intensification in Brazil, employing the SETMI model to estimate ET_a_ and the soil water balance for an intensively grazed elephant grass pasture subjected to intermittent stocking strategies.
- ET_a_ estimated by SETMI using the K_cbrf_ approach well agreed with the values from the EC system. The linear K_cb_-SAVI relationship developed in this study exhibited a strong positive correlation between SAVI and K_cb_, with $\rho$ and ${\mathrm{R}}^{2}$ values of 0.89 and 0.79. This, combined with PlanetScope’s high spatial and temporal resolution imagery, enabled accurate estimation of K_cb_ values during the two-year period.
- By modeling spatial ET_a_ across seasons, we identified periods of water stress and assessed the pasture’s WP, which averaged 2.0 kg m^−3^.
- Our findings highlight the potential of RSWB, ET_a_, and WP assessments to support decision-making, monitoring, and managing water resources in pasture-based livestock farming.

## Supplementary Information

Below is the link to the electronic supplementary material.

Supplementary file1 (DOCX 3410 KB)

## Data Availability

Data will be made available on request.

## References

[CR1] Allen RG, Pereira LS, Howell TA, Jensen ME (2011) Evapotranspiration information reporting: I. Factors governing measurement accuracy. Agric Water Manag 98:899–920. 10.1016/j.agwat.2010.12.015

[CR2] Allen RG, Pereira LS, Raes D, Smith M (1998) Crop evapotranspiration: Guidelines for computing crop water requirements. FAO Irrigation and Drainage Paper No. 56. Food and Agriculture Organization of the United Nations, Rome, Italy

[CR3] Alvares CA, Sentelhas PC, Dias HB (2022) Southeastern Brazil inland tropicalization: Köppen system applied for detecting climate change throughout 100 years of meteorological observed data. Theor Appl Climatol 149:1431–1450. 10.1007/S00704-022-04122-4 35756150 10.1007/s00704-022-04122-4 PMC9213215

[CR4] Alves JDN, Ribeiro A, Rody YP et al (2021) Carbon uptake and water vapor exchange in a pasture site in the Brazilian Cerrado. J Hydrol (Amst) 594:125943. 10.1016/j.jhydrol.2020.125943

[CR5] Alves JDN, Ribeiro A, Rody YP, Loos RA (2022) Energy balance and surface decoupling factor of a pasture in the Brazilian Cerrado. Agric For Meteorol 319:108912. 10.1016/j.agrformet.2022.108912

[CR6] Andrade AS, Santos PM, Pezzopane JRM et al (2016) Simulating tropical forage growth and biomass accumulation: An overview of model development and application. Grass Forage Sci 71:54–65. 10.1111/gfs.12177

[CR7] Barker JB (2017) Spatial irrigation management using remote sensing water balance modeling and soil water content monitoring. University of Nebraska - Lincoln, USA

[CR8] Barker JB, Neale CMU, Heeren DM, Suyker AE (2018) Evaluation of a hybrid reflectance-based crop coefficient and energy balance evapotranspiration model for irrigation management. Trans ASABE 61:533–548

[CR9] Batalha CDA, de Congio GFS, Santos FAP, da Silva SC (2022) Strategic grazing management decreases nitrogen excretion intensity of dairy cows. Sci Agric 79:e20200251. 10.1590/1678-992x-2020-0251

[CR10] Bezerra BG, Santos e Silva CM, Mendes KR et al (2022) CO2 exchanges and evapotranspiration of a grazed pasture under tropical climate conditions. Agric For Meteorol 323:109088. 10.1016/j.agrformet.2022.109088

[CR76] Béziat P, Ceschia E, Dedieu G (2009) Carbon balance of a three crop succession over two cropland sites in South West France. Agric For Meteorol 149:1628–1645. 10.1016/J.AGRFORMET.2009.05.004

[CR11] Bhatti S, Heeren DM, Barker JB et al (2020) Site-specific irrigation management in a sub-humid climate using a spatial evapotranspiration model with satellite and airborne imagery. Agric Water Manag 230:105950. 10.1016/j.agwat.2019.105950

[CR12] Bispo RC, Hernandez FBT, Gonçalves IZ et al (2022) Remote sensing based evapotranspiration modeling for sugarcane in Brazil using a hybrid approach. Agric Water Manag 271:107763. 10.1016/J.AGWAT.2022.107763

[CR13] Botero-Londoño JM, Celis-Celis EM, Botero-Londoño MA (2021) Nutritional quality, nutrient uptake and biomass production of *Pennisetum purpureum* cv. King Grass. Sci Rep 11:13799. 10.1038/s41598-021-93301-w 34226609 10.1038/s41598-021-93301-w PMC8257580

[CR14] Campos I, Neale CMU, Suyker AE et al (2017) Reflectance-based crop coefficients REDUX: For operational evapotranspiration estimates in the age of high producing hybrid varieties. Agric Water Manag 187:140–153. 10.1016/j.agwat.2017.03.022

[CR15] Choudhury BJ, Ahmed NU, Idso SB et al (1994) Relations between evaporation coefficients and vegetation indices studied by model simulations. Remote Sens Environ 50:1–17. 10.1016/0034-4257(94)90090-6

[CR16] Congio GFS, Batalha CDA, Chiavegato MB et al (2018) Strategic grazing management towards sustainable intensification at tropical pasture-based dairy systems. Sci Total Environ 636:872–880. 10.1016/j.scitotenv.2018.04.301 29727853 10.1016/j.scitotenv.2018.04.301

[CR17] Congio GFS (2018) Rotational stocking management on elephant grass for dairy cows: grazing strategies, animal productivity, enteric methane and nitrous oxide emissions. University of Sao Paulo, Luiz de Queiroz College of Agriculture, Brazil

[CR18] Consoli S, Vanella D (2014) Mapping crop evapotranspiration by integrating vegetation indices into a soil water balance model. Agric Water Manag 143:71–81. 10.1016/j.agwat.2014.06.012

[CR19] D’Acunha B, Dalmagro HJ, Zanella de Arruda PH et al (2024) Changes in evapotranspiration, transpiration and evaporation across natural and managed landscapes in the Amazon, Cerrado and Pantanal biomes. Agric for Meteorol 346:109875. 10.1016/j.agrformet.2023.109875

[CR20] da Rocha AEQ, Santos EA, Patrignani A (2022) Partitioning evapotranspiration in a tallgrass prairie using micrometeorological and water use efficiency approaches under contrasting rainfall regimes. J Hydrol (Amst) 608:127624. 10.1016/j.jhydrol.2022.127624

[CR21] da Silva AL, Reichardt K, Roveratti R et al (2007) On the use of soil hydraulic conductivity functions in the field. Soil Tillage Res 93:162–170. 10.1016/j.still.2006.03.024

[CR22] da Silva SC, Sbrissia AF, Pereira LET (2015) Ecophysiology of C4 forage grasses—understanding plant growth for optimising their use and management. Agriculture (Basel) 5:598–625. 10.3390/agriculture5030598

[CR23] da Silva PF, Lima JRdeS, Antonino ACD et al (2017) Seasonal patterns of carbon dioxide, water and energy fluxes over the Caatinga and grassland in the semi-arid region of Brazil. J Arid Environ 147:71–82. 10.1016/J.JARIDENV.2017.09.003

[CR24] da Silva IWH, Marques TV, Urbano SA et al (2024) Meteorological and biophysical controls of evapotranspiration in tropical grazed pasture under rainfed conditions. Agric Water Manag 299:108884. 10.1016/j.agwat.2024.108884

[CR25] da Trindade JK, Carneiro da Silva S, de Souza J, Júnior S et al (2007) Composição morfológica da forragem consumida por bovinos de corte durante o rebaixamento do capim-marandu submetido a estratégias de pastejo rotativo. Pesq Agropec Bras 42:883–890. 10.1590/S0100-204X2007000600016

[CR26] de Oliveira Gonçalves M, Girardi Carpanez T, Batista Gonçalves Silva J et al (2022) Biomass production of the tropical forage grass pennisetum purpureum (BRS Capiaçu) following biofertilizer application. Waste Biomass Valorization 13:2137–2147. 10.1007/s12649-021-01664-y

[CR27] de Oliveira Silva R, Barioni LG, Queiroz Pellegrino G, Moran D (2018) The role of agricultural intensification in Brazil’s Nationally Determined Contribution on emissions mitigation. Agric Syst 161:102–112. 10.1016/j.agsy.2018.01.003

[CR28] Dias LCP, Pimenta FM, Santos AB et al (2016) Patterns of land use, extensification, and intensification of Brazilian agriculture. Glob Chang Biol 22:2887–2903. 10.1111/GCB.13314 27170520 10.1111/gcb.13314

[CR29] Ding R, Kang S, Li F et al (2010) Evaluating eddy covariance method by large-scale weighing lysimeter in a maize field of northwest China. Agric Water Manag 98:87–95. 10.1016/j.agwat.2010.08.001

[CR30] dos Santos ML, Santos PM, Barioni LG et al (2024) Yield gap analysis framework applied to pasture-based livestock systems in Central Brazil. Field Crops Res 314:109416. 10.1016/j.fcr.2024.109416

[CR31] Fisher JB, Melton F, Middleton E et al (2017) The future of evapotranspiration: global requirements for ecosystem functioning, carbon and climate feedbacks, agricultural management, and water resources. Water Resour Res 53:2618–2626. 10.1002/2016WR020175

[CR75] Foken T, Göockede M, Mauder M, Mahrt L, Amiro B, Munger W (2004) Post-field data quality control. In: Handbook of Micrometeorology. pp 181–208. 10.1007/1-4020-2265-4_9

[CR32] Gonçalves IZ, Ruhoff A, Laipelt L et al (2022) Remote sensing-based evapotranspiration modeling using geeSEBAL for sugarcane irrigation management in Brazil. Agric Water Manag 274:107965. 10.1016/J.AGWAT.2022.107965

[CR33] Gonçalves IZ, Mendonça FC, Sanches AC, Marin FR (2024) Optimizing evapotranspiration and crop irrigation requirements of tropical forages cropping systems in Southern Brazil. Int J Biometeorol 68:57–67. 10.1007/s00484-023-02570-9 37880506 10.1007/s00484-023-02570-9

[CR34] Hott MC, Andrade RG, de Magalhães Jr WCP (2024) Distribuição da produção de leite no Brasil. In: ANUÁRIO Leite 2024: avaliação genética multirracial. https://www.embrapa.br/busca-de-publicacoes/-/publicacao/1164754/anuario-leite-2024-avaliacao-genetica-multirracial. Accessed 5 Jun 2025

[CR35] Huete AR (1988) A Soil-Adjusted Vegetation Index (SAVI). Remote Sens Environ 25:295–309. 10.1016/0034-4257(88)90106-X

[CR36] Huete AR, Jackson RD, Post DF (1985) Spectral response of a plant canopy with different soil backgrounds. Remote Sens Environ 17:37–53. 10.1016/0034-4257(85)90111-7

[CR37] Huot C, Zhou Y, Philp JNM, Denton MD (2020) Root depth development in tropical perennial forage grasses is related to root angle, root diameter and leaf area. Plant Soil 456:145–158. 10.1007/s11104-020-04701-2

[CR38] IBGE (2023) Produção da Pecuária Municipal 2023. https://www.ibge.gov.br/estatisticas/economicas/agricultura-e-pecuaria/9107-producao-da-pecuaria-municipal.html?=&t=destaques. Accessed 4 Jun 2025

[CR39] Jin X, Yang G, Xue X et al (2017) Validation of two Huanjing-1A/B satellite-based FAO-56 models for estimating winter wheat crop evapotranspiration during mid-season. Agric Water Manag 189:27–38. 10.1016/j.agwat.2017.04.017

[CR40] Khorchani M, Awada T, Schmer M et al (2024) Long-term croplands water productivity in response to management and climate in the Western US Corn Belt. Agric Water Manag 291:108640. 10.1016/j.agwat.2023.108640

[CR41] Kljun N, Calanca P, Rotach MW, Schmid HP (2015) A simple two-dimensional parameterisation for Flux Footprint Prediction (FFP). Geosci Model Dev 8:3695–3713. 10.5194/GMD-8-3695-2015

[CR42] Majozi NP, Mannaerts CM, Ramoelo A et al (2017) Analysing surface energy balance closure and partitioning over a semi-arid savanna FLUXNET site in Skukuza, Kruger National Park, South Africa. Hydrol Earth Syst Sci 21:3401–3415. 10.5194/hess-21-3401-2017

[CR43] Malafaia GC, Biscola PHN (2025) Anuário CiCarne da cadeia produtiva da carne bovina: 2024–2025. http://www.infoteca.cnptia.embrapa.br/infoteca/handle/doc/1174114. Accessed 10 Jun 2025

[CR44] MapBiomas (2023a) Destaques Agropecuária no Brasil (1985–2022). https://brasil.mapbiomas.org/wp-content/uploads/sites/4/2023/10/FACT_MapBiomas_Agropecuaria_04.10_v2.pdf. Accessed 10 Jun 2025

[CR45] MapBiomas (2023b) Plataforma - MapBiomas Brasil. https://plataforma.brasil.mapbiomas.org/pastagem?activeBaseMap=1&layersOpacity=100&activeModule=quality_of_pasture_data&activeModuleContent=quality_of_pasture_data%3Aquality_of_pasture_data_main&activeYear=2000%2C2022&mapPosition=-10.163560%2C-51.745605%2C5&timelineLimitsRange=2000%2C2022&activeLayers=estados&baseParams[territoryType]=1&baseParams[territories]=1%3BBrasil%3B1%3BPa%C3%ADs%3B0%3B0%3B0%3B0&baseParams[activeClassTreeOptionValue]=quality_of_pasture_main&baseParams[activeClassTreeNodeIds]=136%2C137%2C138&baseParams[activeSubmodule]=quality_of_pasture_data_main&baseParams[yearRange]=1985-2022. Accessed 10 Jun 2025

[CR46] Marin FR, Zanon AJ, Monzon JP et al (2022) Protecting the Amazon forest and reducing global warming via agricultural intensification. Nat Sustain 5:1018–1026. 10.1038/s41893-022-00968-8

[CR47] Meirelles ML, Franco AC, Farias SEM, Bracho R (2011) Evapotranspiration and plant-atmospheric coupling in a *Brachiaria brizantha* pasture in the Brazilian savannah region. Grass Forage Sci 66:206–213. 10.1111/J.1365-2494.2010.00777.X

[CR48] Neale CMU, Bausch WC, Heermann DF et al (1989) Development of reflectance-based crop coefficients for corn. Trans ASAE 32:1891–1899. 10.13031/2013.31240

[CR49] Neale CMU, Geli HME, Kustas WP et al (2012) Soil water content estimation using a remote sensing based hybrid evapotranspiration modeling approach. Adv Water Resour 50:152–161. 10.1016/j.advwatres.2012.10.008

[CR50] Parente L, Ferreira L, Faria A et al (2017) Monitoring the brazilian pasturelands: A new mapping approach based on the landsat 8 spectral and temporal domains. Int J Appl Earth Obs Geoinf 62:135–143. 10.1016/J.JAG.2017.06.003

[CR51] Parente L, Mesquita V, Miziara F et al (2019) Assessing the pasturelands and livestock dynamics in Brazil, from 1985 to 2017: A novel approach based on high spatial resolution imagery and Google Earth Engine cloud computing. Remote Sens Environ 232:111301. 10.1016/J.RSE.2019.111301

[CR52] Pearson K (1896) Mathematical contributions to the theory of evolution.—III. Regression, heredity, and panmixia. Philos Trans R Soc Lond Ser A Contain Pap Math Phys Character 187:253–318. 10.1098/rsta.1896.0007

[CR53] Peel MC, Finlayson BL, Mcmahon TA (2007) Hydrology and Earth System Sciences updated world map of the Köppen-Geiger climate classification. Hydrol Earth Syst Sci 11:1633–1644. 10.5194/hess-11-1633-2007

[CR54] Pereira LET, Paiva AJ, Geremia EV, da Silva SC (2013) Regrowth patterns of elephant grass (*Pennisetum purpureum* Schum) subjected to strategies of intermittent stocking management. Grass Forage Sci 70:195–204. 10.1111/gfs.12103

[CR55] Pereira LET, Paiva AJ, Geremia EV, da Silva SC (2014a) Components of herbage accumulation in elephant grass cvar Napier subjected to strategies of intermittent stocking management. J Agric Sci 152:954–966. 10.1017/S0021859613000695

[CR56] Pereira LET, Paiva AJ, Geremia EV, da Silva SC (2014b) Grazing management and tussock distribution in elephant grass. Grass Forage Sci 70:406–417. 10.1111/gfs.12137

[CR57] Pôças I, Calera A, Campos I, Cunha M (2020) Remote sensing for estimating and mapping single and basal crop coefficients: a review on spectral vegetation indices approaches. Agric Water Manag 233:106081. 10.1016/j.agwat.2020.106081

[CR58] Ribeiro EG, Fontes CAdeA, Palieraqui JGB et al (2008) Influência da irrigação durante as épocas seca e chuvosa na taxa de lotação, no consumo e no desempenho de novilhos em pastagens de capim-elefante e capim-mombaça. Rev Bras Zootec 37:1546–1554. 10.1590/S1516-35982008000900005

[CR59] Santos H, Jacomine PKT, Anjos L (2018) Sistema brasileiro de classificação de solos, 5th edn. Embrapa Solos, Brasília, Brazil

[CR60] Singh BP, Singh HP, Obeng E (2013) Elephantgrass. Biofuel crops: production, physiology and genetics. CABI Publishing, pp 271–291

[CR61] Strassburg BBN, Latawiec AE, Barioni LG et al (2014) When enough should be enough: Improving the use of current agricultural lands could meet production demands and spare natural habitats in Brazil. Glob Environ Chang 28:84–97. 10.1016/j.gloenvcha.2014.06.001

[CR62] Torres EA, Calera A (2010) Bare soil evaporation under high evaporation demand: a proposed modification to the FAO-56 model. Hydrol Sci J 55:303–315. 10.1080/02626661003683249

[CR63] USDA-FAS (2024) Dairy: World Markets and Trade. https://apps.fas.usda.gov/psdonline/circulars/dairy.pdf. Accessed 10 Jun 2025

[CR64] USDA-FAS (2025) Livestock and Poultry: World Markets and Trade. https://apps.fas.usda.gov/psdonline/circulars/livestock_poultry.pdf. Accessed 10 Jun 2025

[CR65] USDA-NRCS (2004) Estimation of Direct Runoff from Storm Rainfall. In: National Engineering Handbook - Part 630 Hydrology. https://irrigationtoolbox.com/NEH/Part630_Hydrology/H_210_630_10.pdf. Accessed 10 Jun 2025

[CR66] van Dijk M, Morley T, Rau ML, Saghai Y (2021) A meta-analysis of projected global food demand and population at risk of hunger for the period 2010–2050. Nat Food 2:494–501. 10.1038/s43016-021-00322-9 37117684 10.1038/s43016-021-00322-9

[CR67] Voltolini TV, Santos FAP, Martinez JC et al (2010) Productive and qualitative characteristics of elephant grass pasture grazed in fixed and intermittent intervals according to interception of active photosynthetic radiation. Revista Brasileira De Zootecnia 39:1002–1010. 10.1590/S1516-35982010000500009

[CR68] Wagle P, Xiao X, Gowda P et al (2017) Analysis and estimation of tallgrass prairie evapotranspiration in the central United States. Agric for Meteorol 232:35–47. 10.1016/j.agrformet.2016.08.005

[CR69] Willmott CJ (1981) On the validation of models. Phys Geogr 2:184–194. 10.1080/02723646.1981.10642213

[CR70] Willmott CJ, Wicks DE (1980) An empirical method for the spatial interpolation of monthly precipitation within California. Phys Geogr 1:59–73. 10.1080/02723646.1980.10642189

[CR71] Wilson JR, Kennedy PM (1996) Plant and animal constraints to voluntary feed intake associated with fibre characteristics and particle breakdown and passage in ruminants. J Agric Res 47:199–225. 10.1071/AR9960199

[CR72] Wutzler T, Lucas-Moffat A, Migliavacca M et al (2018) Basic and extensible post-processing of eddy covariance flux data with REddyProc. Biogeosciences 15:5015–5030. 10.5194/BG-15-5015-2018

[CR73] Zheng H, Yu G, Wang Q et al (2023) Divergent environmental responses of long-term variations in evapotranspiration over four grassland ecosystems in China based on eddy-covariance measurements. J Hydrol (Amst) 625:130030. 10.1016/j.jhydrol.2023.130030

[CR74] Zou KH, Tuncali K, Silverman SG (2003) Correlation and simple linear regression. Radiology 227:617–622. 10.1148/radiol.2273011499 12773666 10.1148/radiol.2273011499

