# Supplementary material for: Remote sensing-based evapotranspiration and soil water balance estimation for a tropical pasture in Brazil using the SETMI model
Source: Int J Biometeorol. 2026 Jun 30;70(7):198. doi: 10.1007/s00484-026-03255-9 (PMC13319695; doi:10.1007/s00484-026-03255-9)
Supplement: Supplementary file 1 — Supplementary file1 (DOCX 3410 KB) [file 484_2026_3255_MOESM1_ESM.docx]

**Supplementary material**

**Remote sensing-based evapotranspiration and soil water balance estimation for a tropical pasture in Brazil using the SETMI model**

Vitor de J. M. Bianchini^a^*, Ivo Z. Gonçalves^b^, Christopher M. U. Neale^b^, Alex da S. Sechi^a^, Thieres G. F. da Silva^c^, Fábio R. Marin^a^

^a^ Department of Biosystems Engineering, Luiz de Queiroz College of Agriculture, University of São Paulo, Piracicaba, SP, Brazil

^b^ Daugherty Water for Food Global Institute, University of Nebraska-Lincoln, Lincoln, NE, USA

^c^ Department of Agricultural Engineering, Federal Rural University of Pernambuco, Recife, PE, Brazil

*Corresponding author (VJM Bianchini). E-mail: [vitor.bianchini@usp.br](mailto:vitor.bianchini@usp.br)


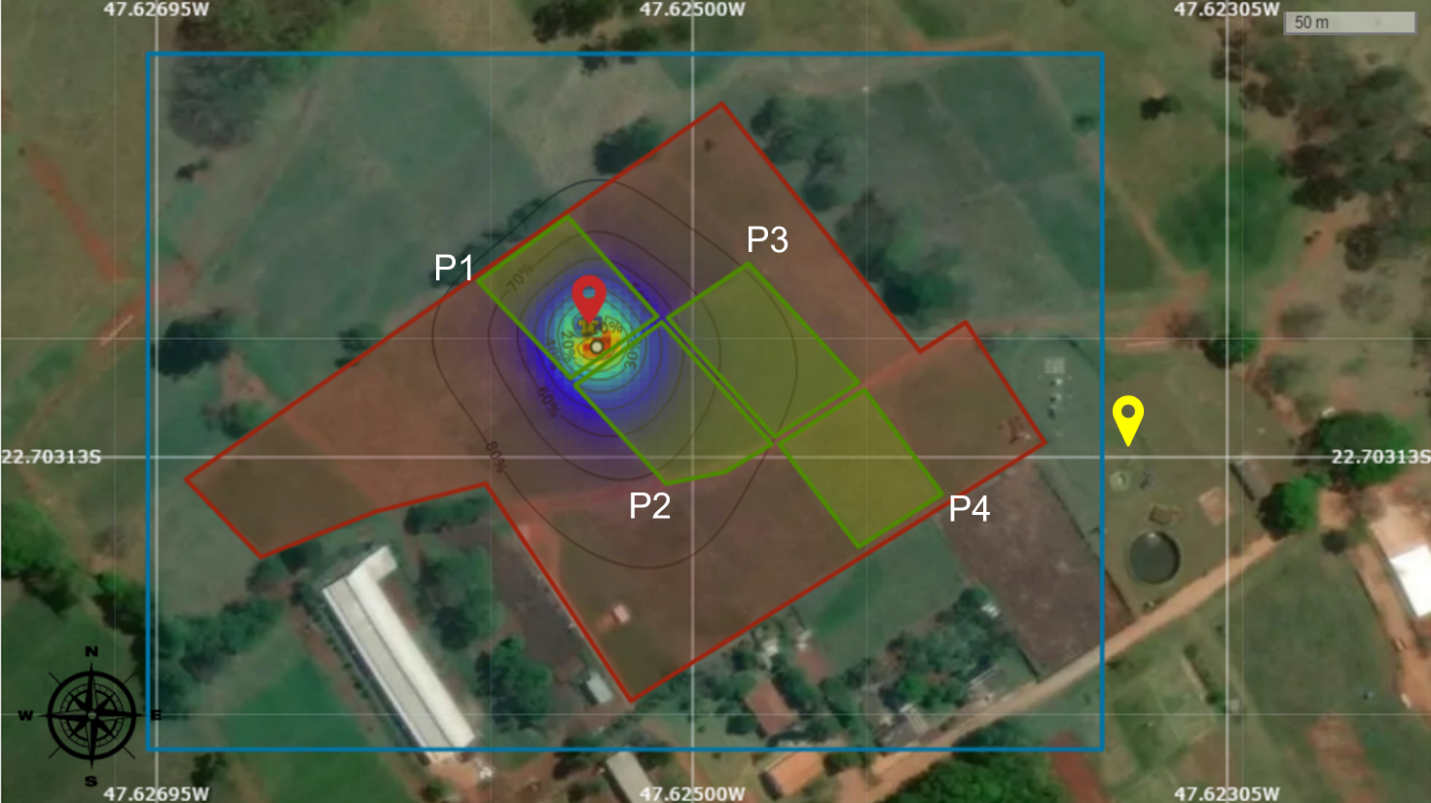


**Fig. S1.** Illustration of the experimental site located at the Luiz de Queiroz College of Agriculture (Esalq). The red marker indicates the location of the Eddy Covariance tower. The contours and the heat map surrounding the red marker represent the tower's footprint. The area outlined in red corresponds to the elephant grass pasture under rational grazing. The areas outlined in green (P1 - P4) are the paddocks where the leaf area index and herbage mass were measured. The yellow marker indicates the location of the Esalq weather station.


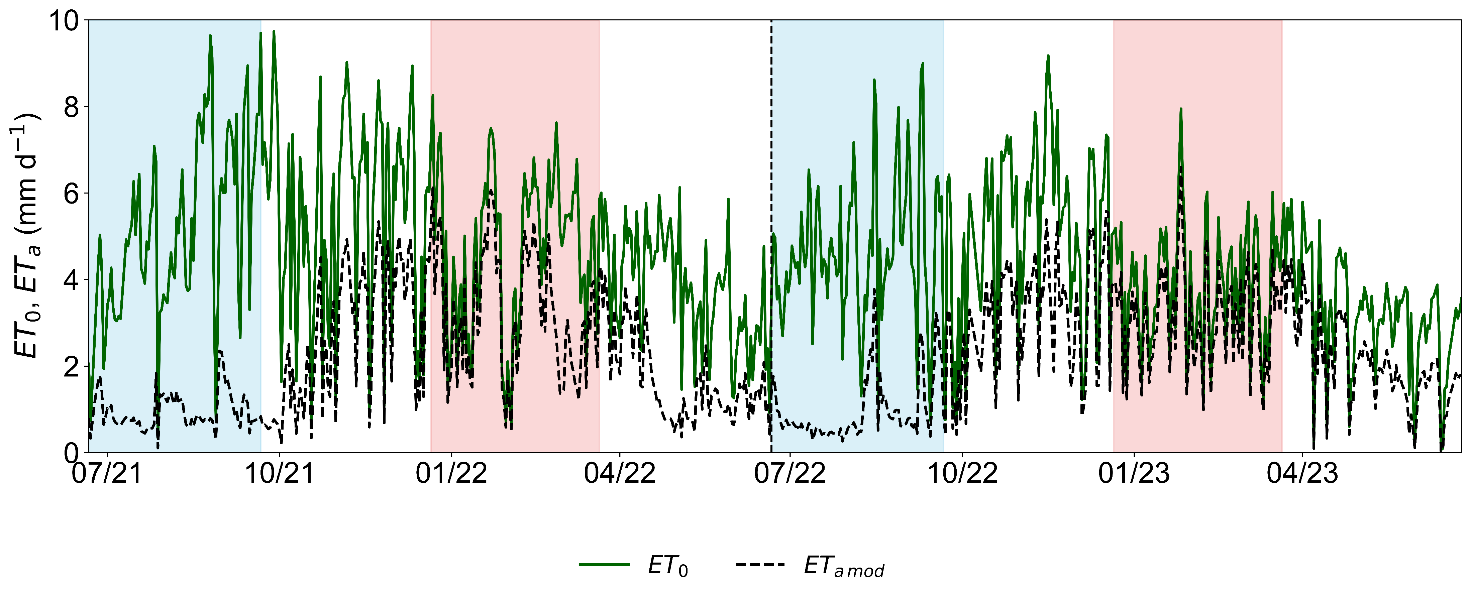


**Fig. S2.** Daily reference evapotranspiration (ET_0_) and modeled actual evapotranspiration (ET_a mod_). The blue-shaded regions represent the winters, and the red-shaded regions the summers. The unshaded (white) regions correspond to the transitional seasons: spring and fall. The vertical dashed line separates the 2021-2022 (left) and 2022-2023 (right) periods.


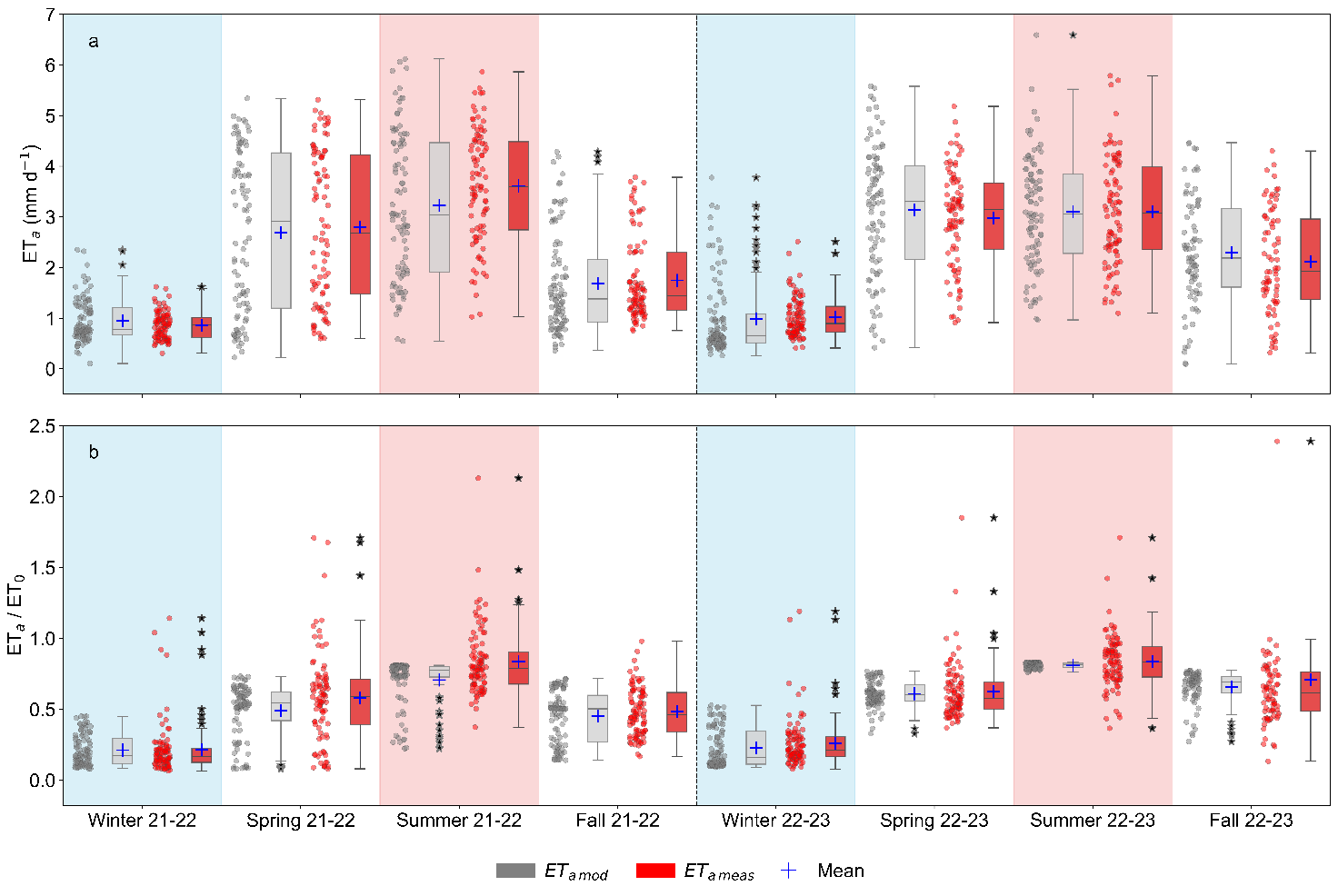


**Fig. S3.** Seasonal variation of (a) modeled (ET_a mod_) and measured (ET_a meas_) actual evapotranspiration and (b) ET_a_ / ET_0_ ratio. The blue-shaded regions represent the winters, and the red-shaded regions the summers. The unshaded (white) regions correspond to the transitional seasons: spring and fall. The vertical dashed line separates the 2021-2022 (left) and 2022-2023 (right) periods.

**Table S1.** Summary of selected meteorological variables and evapotranspiration.

|  | 2021-2022 | | | |  | 2022-2023 | | | |  |
| --- | --- | --- | --- | --- | --- | --- | --- | --- | --- | --- |
| **Variable** | Winter | Spring | Summer | Fall | **Annual 2021-2022** | Winter | Spring | Summer | Fall | **Annual 2022-2023** |
| **T_max_ (ºC)** | 28.7 ± 5.0 | 30.4 ± 3.6 | 30.9 ± 2.9 | 27.5 ± 3.8 | **29.4 ± 4.1** | 27.7 ± 3.7 | 29.2 ± 3.7 | 30.0 ± 2.3 | 27.2 ± 3.5 | **28.5 ± 3.5** |
| **T_min_ (ºC)** | 11.7 ± 4.4 | 17.0 ± 2.2 | 19.5 ± 1.4 | 14.4 ± 4.3 | **15.6 ± 4.4** | 12.3 ± 2.4 | 16.8 ± 2.6 | 19.3 ± 1.1 | 14.2 ± 3.9 | **15.6 ± 3.8** |
| **T_avg_ (ºC)** | 20.2 ± 4.2 | 23.7 ± 2.2 | 25.2 ± 1.8 | 20.9 ± 3.6 | **22.5 ± 3.7** | 20.0 ± 2.4 | 23.0 ± 2.6 | 24.7 ± 1.4 | 20.7 ± 3.2 | **22.1 ± 3.1** |
| **VPD (kPa)** | 0.97 ± 0.47 | 0.92 ± 0.42 | 0.76 ± 0.33 | 0.66 ± 0.22 | **0.83 ± 0.39** | 0.85 ± 0.32 | 0.80 ± 0.34 | 0.54 ± 0.23 | 0.55 ± 0.22 | **0.69 ± 0.31** |
| **Ppt (mm)** | 45 | 347 | 718 | 98 | **1208** | 61 | 327 | 786 | 227 | **1403** |
| **ET_0_ (mm)** | 468 | 514 | 422 | 348 | **1751** | 428 | 463 | 345 | 299 | **1534** |
| **ET_a meas_ (mm)** | 79 | 248 | 328 | 161 | **817** | 92 | 265 | 278 | 160 | **796** |
| **ET_a mod_ (mm)** | 89 | 240 | 290 | 154 | **775** | 93 | 280 | 279 | 169 | **821** |

T_max_: average maximum temperature; T_min_: average minimum temperature; T_avg_: average temperature, VPD: vapor pressure deficit; ET_0_: reference evapotranspiration; Ppt: gross precipitation; ET_a mod_: actual evapotranspiration modelled using SETMI; ET_a meas_: actual evapotranspiration measured by the eddy covariance system.
